# Can we monitor heart attack in the troponin era: evidence from a population-based cohort study

**DOI:** 10.1186/1471-2261-11-35

**Published:** 2011-06-24

**Authors:** Frank M Sanfilippo, Michael ST Hobbs, Matthew W Knuiman, Stephen C Ridout, Pamela J Bradshaw, Judith C Finn, Jamie M Rankin, Peter C Sprivulis, Joseph Hung

**Affiliations:** 1 School of Population Health M431, University of Western Australia, 35 Stirling Highway, Crawley 6009 WA, Australia; 2 Discipline of Emergency Medicine M516, School of Primary, Aboriginal & Rural Health Care, University of Western Australia, 35 Stirling Highway, Crawley 6009 WA, Australia; 3 Department of Cardiology, Royal Perth Hospital, Wellington Street, Perth 6000 WA, Australia; 4 Department of Emergency Medicine, Fremantle Hospital, Alma Street, Fremantle 6160 WA, Australia; 5 School of Medicine and Pharmacology M503, University of Western Australia, 35 Stirling Highway, Crawley 6009 WA, Australia; 6 Cardiology Department, Sir Charles Gairdner Hospital, Hospital Avenue, Nedlands 6009 WA, Australia

**Keywords:** AHA Medical/Scientific Statements, cardiac biomarkers, diagnosis, myocardial infarction, trends

## Abstract

**Background:**

Troponins (highly sensitive biomarkers of myocardial damage) increase counts of myocardial infarction (MI) in clinical practice, but their impact on trends in admission rates for MI in National statistics is uncertain.

**Methods:**

Cases coded as MI or other cardiac diagnoses in the Hospital Morbidity Data Collection (MI-HMDC) in Western Australia in 1998 and 2003 were classified using revised criteria for MI developed by an International panel convened by the American Heart Association (AHA criteria) using information on symptoms, ECGs and cardiac biomarkers abstracted from samples of medical notes. Age-sex standardized rates of MI-HMDC were compared with rates of MI based on AHA criteria including troponins (MI-AHA) or traditional biomarkers only (MI-AHAck).

**Results:**

Between 1998 and 2003, rates of MI-HMDC decreased by 3.5% whereas rates of MI-AHA increased by 17%, a difference largely due to increased false-negative cases in the HMDC associated with marked increased use of troponin tests in cardiac admissions generally, and progressively lower test thresholds. In contrast, rates of MI-AHA_ck _declined by 18%.

**Conclusions:**

Increasing misclassification of MI-AHA by the HMDC may be due to reluctance by clinicians to diagnose MI based on relatively small increases in troponin levels. These influences are likely to continue. Monitoring MI using AHA criteria will require calibration of commercially available troponin tests and agreement on lower diagnostic thresholds for epidemiological studies. Declining rates of MI-AHA_ck _are consistent with long-standing trends in MI in Western Australia, suggesting that neither MI-HMDC nor MI-AHA reflect the true underlying population trends in MI.

## Background

Coronary heart disease (CHD), despite declining mortality, remains a major health problem in developed countries [1-3]. Reliable methods for monitoring acute CHD events, including myocardial infarction (MI), are therefore essential for evaluation of preventive and clinical services, particularly in view of the increasing prevalence of obesity and diabetes that could diminish or reverse the favourable trends in mortality. The World Health Organization (WHO) MONICA Project, which monitored trends and determinants of coronary heart disease including non-fatal MI for 10 years in 25 countries between 1983 and 1995, demonstrated the importance of population-based registers to monitor MI, using standardised methods of case-finding and unchanging diagnostic criteria. Unfortunately, such registers are costly and there have been few recent national or international register-based studies of trends in MI [4-7].

In many jurisdictions, routinely collected mortality and hospital morbidity data are therefore the most commonly used alternative for monitoring trends in MI. While such data have major shortcomings, studies in Finland, Sweden and Western Australia have previously shown reasonable agreement between trends based on registers and administrative data [7-9]. However, the recent widespread introduction into clinical practice of highly sensitive and specific biomarkers of myocardial damage, particularly troponin tests, raises doubts about the reliability of administrative data for monitoring MI. Several studies have demonstrated greatly increased counts of MI using troponin tests compared with traditional biomarkers [4,10]. Studies in Perth, Western Australia, and Denmark have shown that declining trends in hospital MI admissions reversed or levelled out since the introduction of troponin tests in 1996 [5,11].

In recognition of the potential problems for epidemiological studies, a panel of international experts, meeting under the auspices of the American Heart Association (AHA), have developed new criteria for MI for use in epidemiological studies, referred to here as the 'AHA criteria', which emphasise the importance of troponin in the diagnosis of MI [12]. So far, there have been few population-based studies exploring the practical issues of implementing the new criteria or assessing the impact of troponin tests on trends in MI based on administrative data.

This study used the Hospital Morbidity Data Collection (HMDC), one of the core administrative datasets in the Western Australian Data Linkage System [13], to compare counts of non-fatal MI (MI-HMDC) in 1998 and 2003 with counts based on AHA criteria using all biomarkers including troponins (MI-AHA), or traditional biomarkers only (MI-AHA_ck_) including creatinine kinase (CK) or CK-MB in the classification algorithm.

## Methods

The study population consisted of residents of the Perth Statistical Division (population 1.43 million in 2003) of Western Australia aged 35-79 years admitted to hospital in 1998 or 2003 for cardiac conditions or chest pain (International Classification of Diseases (ICD) 9^th ^revision codes 401-429, 786.5; or ICD10 Australian Modification codes I10-I52, R07). Electronic records for the study population were extracted from the HMDC, identified by hierarchical discharge diagnosis as shown in Table 1, then linked to provide 28-day episodes of care.

**Table 1:** Hierarchical classification of cardiovascular discharge diagnoses in non-fatal cases which identified the validation population and sample


**Diagnosis**	**ICD codes**		**1998**		**2003**	
	
	**ICD-9-CM**	**ICD-10-AM**	**Counts** **(n = 8939)**	**% Troponin tests**^**a**^	**Counts** **(n = 9188)**	**% Troponin tests**^**a**^

MI^b^	410	I21, I22	1425	81	1582	97
Unstable angina^b^	411.1	I20.0	2024	53	1507	93
Other IHD^c^						
Other Angina	other 411, 413	other I20	620	37	548	75
Other IHD	rest of 410-414	I24, I25	135	15	216	54
Other CVD^c^						
Heart Failure	428	I50	820	32	655	79
Valve Disorders	424.0, 424.1	I34, I35	59	19	66	29
Arrhythmia	427	I46-I49	1557	21	1723	46
Hypertension	401-405	I10-I15	159	4	100	30
Other cardiovascular	rest of 401-429	rest of I10-I52	515	20	514	51
Chest pain^c^	786.5	R07	1625	46	2277	82

Cases were based on 28-day episodes of care. CVD: cardiovascular disease; ICD: International Classification of Diseases; IHD: ischaemic heart disease.

^a ^% troponin tests = proportion of cases who had at least one troponin test.

^b ^From any diagnosis field of the HMDC (Hospital Morbidity Data Collection).

^c ^From principal diagnosis field of the HMDC.

Non-fatal cases were defined as patients who were alive 28 days after admission. The episode records were then linked electronically to results of cardiac biomarkers provided by biochemistry departments. Cases were then selected for validation of discharge diagnosis codes against information abstracted from medical notes, using the sampling scheme outlined in Additional File 1, Table S1. In brief, cases for validation consisted of random samples of all non-fatal cases coded as MI or unstable angina pectoris (UAP) in any diagnosis field, or cases with a principal diagnosis of other heart disease or chest pain who had positive biomarker test results (suspected false negative MI). We excluded booked admissions for coronary artery bypass surgery or heart valve operations; angiograms with length of stay ≤ 1 day; and heart transplants.

Data were abstracted from medical notes directly into a Microsoft Access database by trained staff using a standardised data collection format. Data included: symptoms present on admission, results of biomarker tests (daily results for up to 5 days), reasons for possible false elevations of cardiac biomarkers (such as angioplasty, cardiac surgery, other major surgery, trauma, severe renal failure), and photocopies of up to five electrocardiographs (ECG). Other data, including demographic details and dates, were added directly from the HMDC extract.

### Classification of myocardial infarction by 'AHA criteria'

A computer algorithm was developed to classify cases as Definite, Probable, Possible or Not MI according to AHA criteria based on the combination of symptoms, biomarker results and ECG abnormalities as defined in the International panel report [12] and illustrated in Additional File 1, Table S2.

### Statistical analyses

All analyses were carried out in SAS version 9.1.3 SP4 for Windows. Population estimates of the total number of cases in each sample group were calculated by inflating the sampled cases by their sampling fraction (see Additional File 1, Table S1). This was achieved using the inverse of the sampling fraction as the variable in the weight statement of Proc Freq in SAS, and in Proc SurveyFreq when calculating sensitivity and PPV. Results are reported as counts and proportions, together with population estimates of sensitivity, positive predictive value (PPV) and their 95% confidence intervals for the HMDC coding of MI relative to 'AHA criteria' for Definite/Probable MI (Positive MI) and Definite/Probable/Possible MI (Any MI). The percentage by which HMDC misclassified MI as defined by 'AHA criteria' was calculated by .

Counts of MI-HMDC were compared separately with counts of MI based on AHA criteria using all available biomarkers, including troponins (MI-AHA), or traditional biomarkers only (MI-AHA_ck_). To allow for the effect of population increase between 1998 and 2003, we estimated age-sex standardised rates of admission for non-fatal MI in age group 35-79 years using the direct method by 5-year age group and sex, with the Australian estimated population at 30 June 2001 as the standard.

Diagnostic thresholds for troponin tests were lower in 2003 than in 1998 as the sensitivity of the assays improved. We compared the distribution of ECG changes and biomarker results in cases of Positive MI coded in the HMDC as MI (true-positives) or Not MI (false-negatives) to examine the extent to which the lower thresholds were associated with false-negative cases.

### Ethics approval

This study was approved by the Human Research Ethics Committees of the University of Western Australia, each of the eight hospitals from which data were collected, and by the Western Australian Confidentiality of Health Information Committee. The study was granted a waiver of informed consent.

## Results

In 1998, there were 8939 28-day episodes of care for heart conditions or chest pain (Table 1) of which 3522 met our criteria for validation (see Additional File 1, Table S1) and from which 1456 non-fatal episodes were sampled for validation against medical notes. The equivalent numbers for 2003 were 9188 episodes of care, 3297 meeting validation criteria and 1108 sampled for validation. Between 1998 and 2003, episodes of care for MI increased by 11% whilst those for UAP decreased by 26%. In 1998, 81% of episodes of care for MI and 53% of UAP had troponin tests, increasing to 97% and 93% respectively in 2003. In comparison, the traditional biomarkers (CK and CK-MB) were used in 96% of episodes of MI care in 1998 and 93% in 2003. The prevalence of troponin tests in cases not coded as MI or UAP increased from 33% to 63%.

### AHA classification scheme

Full details of the final AHA diagnostic classification of validated cases together with corresponding classifications of symptoms, ECG and biomarker results for 1998 and 2003 are shown in Additional File 1, Table S2. A feature of this is the dominating influence of biomarkers, particularly troponins, on the classification of MI. For example, in 1998, 74.8% of cases were classified as Definite MI because of biomarkers alone, whilst only 6.8% were classified as Definite MI based on evolving ECG changes alone. The corresponding values in 2003 were 81.1% (1342/1654) and 8.3% (138/1654).

### Comparison of MI-HMDC and MI-AHA

Table 2 shows the AHA classification of MI cross-tabulated by HMDC diagnosis. As there were few cases of AHA Probable MI in either year (1.2% in 1998 and 3.5% in 2003), these were combined with Definite MI in all tables (Positive MI). The sensitivity, positive predictive value (PPV) and estimated misclassification of MI-HMDC for Positive MI and Any MI are shown in Table 3. In 1998, MI-HMDC overestimated Positive MI by 7.4% but underestimated this by 10.7% in 2003. Any MI (Positive + Possible MI) was underestimated by 12.4% in 1998 and by 21.3% in 2003. These temporal changes in misclassification between 1998 and 2003 resulted from general improvement in PPV (from 77.3% to 83.5% for Positive MI) but deteriorating sensitivity (from 82.9% to 74.6%). For example, in 1998, 12.3% of cases classified as Positive MI were coded as UAP in the HMDC (Table 2), and 4.8% as other heart diseases or chest pain, but in 2003 the respective proportions were 15.1% for UAP and 10.3% for other cardiac conditions. When conditions other than ischemic heart disease were excluded from the analysis, the sensitivity of MI-HMDC increased to 86.1% from 82.9% in 1998, and to 80.8% from 74.6% in 2003.

**Table 2:** Hierarchical diagnosis and classification of myocardial infarction based on AHA Criteria for non-fatal population estimates


**Year**	**HMDC episodes of care**		**AHA classification of MI**^**a **^**n (row%, col%)**		
	
	**Hierarchical diagnosis**	**Count**	**Positive MI**	**Any MI**	**Not MI**

1998	MI	1420	1098 (77.3, 82.9)	1309 (92.2, 80.8)	111 (7.8, 5.8)
	Unstable angina	2011	163 (8.1, 12.3)	233 (11.6, 14.4)	1778 (88.4, 93.5)
	Other IHD^b^	22	14 (63.6, 1.1)	20 (90.9, 1.2)	2 (9.1, 0.1)
	Other CVD^b^	69	49 (71.0, 3.7)	58 (84.1, 3.6)	11 (15.9, 0.6)
	**Total**	**3522**	**1324**	**1620 **^c^	**1902**
2003	MI	1579	1318 (83.5, 74.6)	1479 (93.7, 73.8)	100 (6.3, 7.7)
	Unstable angina	1493	267 (17.9, 15.1)	329 (22.0, 16.4)	1164 (78.0, 90.1)
	Other IHD^b^	48	46 (95.8, 2.6)	46 (95.8, 2.3)	2 (4.2, 0.2)
	Other CVD^b^	177	137 (77.4, 7.7)	151 (85.3, 7.5)	26 (14.7, 2.0)
	**Total**	**3297**	**1768**	**2005 **^c^	**1292**

AHA: American Heart Association; CVD: cardiovascular disease; IHD: ischaemic heart disease; MI: myocardial infarction; Positive MI: AHA Definite or Probable MI; Any MI: AHA Definite/Probable/Possible MI.

^a ^Luepker et al [12]. Row% represents positive predictive value, col% represents sensitivity.

^b ^As listed in Table 1 (Other CVD includes chest pain).

^c ^Includes 66 cases (1998) and 64 cases (2003) downgraded from Definite due to absence of diagnostic troponin test.

**Table 3:** Sensitivity and PPV of MI-HMDC for non-fatal population estimates based on AHA classification of myocardial infarction


**Group**	**1998**					**2003**				
		
	**Sn, 95% CI**		**PPV, 95% CI**		**Sn/PPV **^**a**^	**Sn, 95% CI**		**PPV, 95% CI**		**Sn/PPV **^**a**^

**Positive MI^b^**										
Total non-fatal	82.9	80.3, 85.6	77.3	74.2, 80.4	1.07 (+7.4)	74.6	72.9, 76.2	83.5	79.4, 87.6	0.89 (-10.7)
All males	83.2	80.2, 86.3	76.6	72.9, 80.3	1.09 (+8.6)	76.5	74.6, 78.3	85.6	81.0, 90.2	0.89 (-10.6)
All females	82.2	77.0, 87.3	79.1	73.4, 84.8	1.04 (+3.9)	69.6	65.7, 73.4	77.9	69.1, 86.8	0.89 (-10.7)
All 35-69 years	83.8	80.3, 87.4	81.6	78.0, 85.2	1.03 (+2.7)	77.4	75.3, 79.6	85.0	80.1, 89.9	0.91 (-8.9)
All 70-79 years	81.2	77.2, 85.3	70.0	64.4, 75.6	1.16 (+16.0)	69.9	67.0, 72.8	80.6	73.0, 88.1	0.87 (-13.3)

**Any MI^b^**										
Total non-fatal	80.8	78.5, 83.1	92.2	90.2, 94.2	0.88 (-12.4)	73.8	72.2, 75.2	93.7	90.9, 96.4	0.79 (-21.3)
All males	80.5	77.9, 83.2	92.1	89.7, 94.4	0.87 (-12.6)	76.2	74.6, 77.7	94.3	(91.3, 97.3	0.81 (-19.2)
All females	81.6	76.9, 86.2	92.5	88.9, 96.2	0.88 (-11.8)	67.7	64.2, 71.2	91.9	86.0, 97.7	0.74 (-26.3)
All 35-69 years	80.7	77.5, 83.9	94.2	92.0, 96.4	0.86 (-14.3)	76.9	75.0, 78.8	94.7	91.6, 97.8	0.81 (-18.8)
All 70-79 years	80.8	77.6, 84.1	88.7	84.8, 92.6	0.91 (-8.9)	68.9	66.4, 71.3	91.7	86.4, 96.9	0.75 (-24.9)

95% CI: 95% confidence interval; AHA: American Heart Association; HMDC: Hospital Morbidity Data Collection; MI: myocardial infarction; MI-HMDC: MI coded in the HMDC; Positive MI: AHA Definite or Probable MI; Any MI: AHA Definite/Probable/Possible MI; PPV: positive predictive value; Sn: sensitivity.

^a ^Ratio of sensitivity to PPV: number in parentheses represents the percentage by which the HMDC over-estimates (+) or under-estimates (-) the number of cases of MI in the population as defined by the 'AHA criteria'.

^b ^Luepker et al [12].

Table 3 also demonstrates some variation in PPV and sensitivity of MI-HMDC by broad age group and sex, but there was no consistent pattern except for generally lower PPV and sensitivity in the 70-79 year age group, and the variation could be due to chance as seen through the 95% confidence intervals.

### Characteristics of false-negative cases

**Figure 1:**
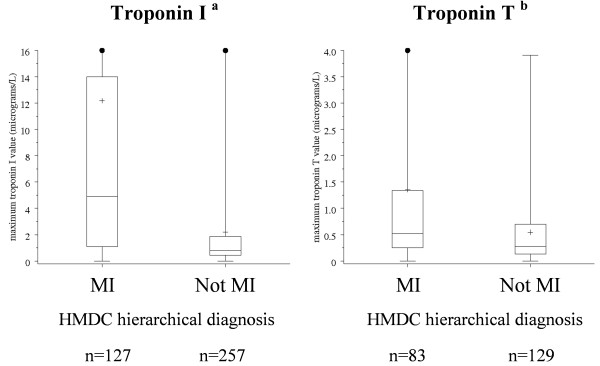
**Box plots of distribution of maximum values for troponin I and T according to HMDC coding of MI or not MI in cases classified as AHA Definite or Probable MI (Positive MI) based on positive troponin tests but non-specific or normal ECGs in the 2003 validation sample**. + indicates mean value of distribution; • indicates extreme values beyond the y-axis scale. MI-HMDC represents true-positives and HMDC Not MI represents false-negatives. HMDC: Hospital Morbidity Data Collection; MI: myocardial infarction. ^a ^p < 0.0001 and ^b ^p = 0.0006 for MI vs not MI (two-sided Wilcoxon rank sum test with normal approximation).

To understand the declining sensitivity of MI-HMDC between 1998 and 2003 we studied the distributions of ECG changes and biomarker results in false-negative cases (AHA Positive MI, but not coded as MI in the HMDC). In 1998, 49% of MI-HMDC had ECG evidence of MI (diagnostic or positive ECGs), compared with 31% in false-negative cases. In 2003, the corresponding percentages were 42% and 23%. Conversely, in both years 28% of MI-HMDC had normal/other ECGs compared with around 45% in false-negative cases. We also found that troponin levels were substantially lower in false-negative cases than in true-positive cases as illustrated in Figure 1 which compares the distribution of troponin levels in true-positive and false-negative cases based on AHA Positive MI in 2003.

### Comparison of counts of MI based on troponins or traditional biomarkers

**Figure 2:**
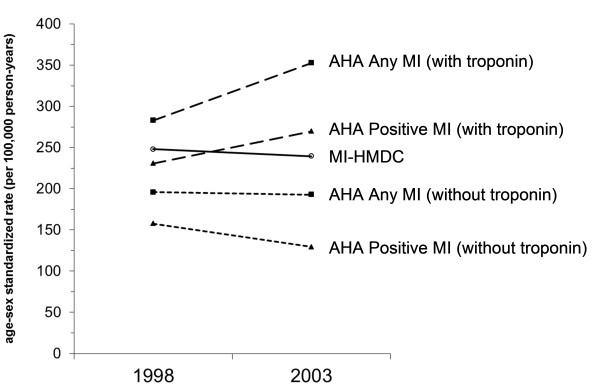
**Trends in age-sex standardized admission rates for non-fatal MI in age group 35-79 years as recorded in the HMDC compared with AHA classification of MI**. AHA: American Heart Association; HMDC: Hospital Morbidity Data Collection; MI: myocardial infarction; Any MI: AHA Definite/Probable/Possible MI; Positive MI: AHA Definite or Probable MI; MI-HMDC: MI coded in the HMDC.

Table 4 shows the AHA classification by HMDC diagnosis when troponin tests are excluded from the classification algorithm. Compared with Table 2, this shows that troponin tests increased the number of Positive MI cases from 913 to 1324 in 1998 (45% increase) and from 853 to 1768 in 2003 (107% increase). For Any MI, troponin tests were associated with 43% more cases in 1998 and 82% more cases in 2003. Figure 2 demonstrates these changes in counts as age-sex standardised rates to allow for population increase. Between 1998 and 2003, there was a small decline (3.5%) in age-sex standardised rates of admission for non-fatal MI-HMDC. In contrast, rates of Positive MI (with troponin) increased by 17% while rates of Positive MI (without troponin) declined by 18%. Rates of Any MI (with troponin) increased by 24% while rates of Any MI (without troponin) declined by 1.5%.

**Table 4:** Hierarchical diagnosis and classification of myocardial infarction based on AHA Criteria using only CK biomarkers


**Year**	**HMDC episodes of care**		**AHA classification of MI**^**a **^**n (row%, col%)**		
	
	**Hierarchical diagnosis**	**Count**	**Positive MI**	**Any MI**	**Not MI**

1998	MI	1420	816 (57.5, 89.4)	999 (70.4, 88.5)	421 (29.6, 17.6)
	Unstable angina	2011	89 (4.4, 9.8)	115 (5.7, 10.2)	1896 (94.3, 79.2)
	Other IHD^b^	22	4 (18.2, 0.4)	4 (18.2, 0.3)	18 (81.8, 0.8)
	Other CVD^b^	69	4 (5.8, 0.4)	11 (15.9, 1.0)	58 (84.1, 2.4)
	**Total**	**3522**	**913**	**1129**	**2393**
2003	MI	1579	752 (47.6, 88.2)	962 (60.9, 87.1)	617 (39.1, 28.1)
	Unstable angina	1493	67 (4.5, 7.8)	99 (6.6, 9.0)	1394 (93.4, 63.6)
	Other IHD^b^	48	11 (22.9, 1.3)	13 (27.1, 1.2)	35 (72.9, 1.6)
	Other CVD^b^	177	23 (13.0, 2.7)	30 (17.0, 2.7)	147 (83.0, 6.7)
	**Total**	**3297**	**853**	**1104**	**2193**

For non-fatal population estimates in 1998 and 2003 using only CK or CK-MB biomarker results. AHA: American Heart Association; CK: creatinine kinase; CK-MB: MB isoenzyme of CK; CVD: cardiovascular disease; HMDC: Hospital Morbidity Data Collection; MI: myocardial infarction; MI-HMDC: MI coded in the HMDC; Positive MI: AHA Definite or Probable MI; Any MI: AHA Definite/Probable/Possible MI; IHD: ischaemic heart disease.

^a ^Luepker et al [12]. Row% represents positive predictive value, col% represents sensitivity.

^b ^As listed in Table 1 (Other CVD includes chest pain).

## Discussion

The introduction of troponins as highly sensitive and specific diagnostic tests for MI has revolutionised the clinical management of suspected myocardial infarction, but while their utility in clinical practice is unquestioned, problems in monitoring population trends in MI remain unresolved. We have made a three-way comparison of counts of MI-HMDC, MI-AHA (with troponin) and MI-AHA_ck _(without troponin). When troponin is included in the AHA classification, MI-HMDC overestimated counts of Positive MI in 1998 but under-estimated the counts in 2003. This disparity between the clinical (coded) diagnosis in 2003 is in agreement with the finding by Roger and colleagues in their prospective study of the effects of troponin tests on counts of MI in Olmsted County, that found substantially lower counts of cases with a final coded diagnosis of MI in medical records compared with counts based on troponin tests [10]. They attributed this to a reluctance by clinicians to always accept relatively low levels of troponin as diagnostic of MI.

In contrast, when troponin was excluded from the AHA classification, MI-HMDC overestimated counts of Positive MI which actually decreased between 1998 and 2003. There was an even greater difference between the increasing rates of MI-AHA (with troponin) and the decreasing rates of MI-AHA_ck _(without troponin). The decrease in MI-AHA_ck _is consistent with the decline in admission rates for MI in Perth in the 10 years prior to the introduction of troponin tests [11]. The marked divergence between MI-AHA (with troponin) and MI-AHA_ck _is also consistent with a further study in Olmsted County that found that from 1987 to 2006, rates of MI declined by 20% when traditional biomarkers only were used in the diagnosis of MI, whereas rates of MI increased when based on troponin tests [14].

An essential requirement for monitoring MI is that classification should be based on objective criteria that remain constant over time. In the case of troponin-based criteria this is unlikely because of progressive lowering of diagnostic thresholds as the precision of tests improves, and because of variation within and between hospitals in the several commercial, non-standardised tests in use. This is likely to increase the number of false negative cases. A further likely reason for the increase in false-negative cases in 2003 was the marked general increase (from 33 to 66%) in use of troponin tests in all cardiac admissions other than MI or UAP. Thus, even though the prevalence of positive troponin tests in such cases was small, it had a relatively large negative impact on the sensitivity of MI-HMDC against MI-AHA.

### Strengths of the study

Western Australia is geographically isolated with relatively small population losses from emigration, and is thus ideal for epidemiological studies. It has comprehensive, linked health statistics systems spanning 30 years [13], and has been the site of several previous studies of trends in MI, including the WHO MONICA Project [9,13,15]. Record linkage allowed us to define 28-day episodes of care, thus eliminating inflation due to transfers and early readmissions, and provided a total population sampling frame for validation studies. Direct linkage of HMDC records to laboratory records of biomarker results allowed us to identify efficiently any potential false-negative cases of MI. Finally, despite the rapid uptake of troponin tests in Perth, the continued high use of CK tests in the diagnosis of MI allowed us to classify cases using both new and traditional biomarkers, providing a direct measure of the impact of troponin tests on the diagnosis of MI in administrative data over time.

### Limitations

Limited resources forced us to adopt a sampling strategy to validate the coding of MI in the HMDC. If sampling was not strictly random, errors may have occurred in population estimates. Our strategy of linking biomarker test results to identify possible false-negative cases of MI in the HMDC may have identified some cases in which elevated troponins were not due to an acute ischaemic event (for example, in chronic heart failure) but which nevertheless met the 'AHA criteria' for MI. Lack of statistical power due to limited resources also prevented us from fully exploring possible differences in the impact of troponins on rates of MI by age and gender.

## Conclusions

Our study has identified a number of issues for further investigation before the predominately troponin-based 'AHA criteria' can be used with confidence in studies of trends in MI. The most urgent need is for calibration of the several commercial troponin tests in use, and for agreement on stratification of troponin results that would permit valid comparison of positive tests over time, despite changing diagnostic thresholds. While universal standardization of troponin tests is unlikely ever to be possible, calibration for studies of trends at regional level, particularly when only a few laboratories are involved, should be possible. This would not invalidate comparative studies within or between countries in which the primary focus is on trends rather than cross-sectional differences between rates, and providing that the methods of calibration are explicitly described.

In view of the marked general increases in the use of troponin tests in acutely ill patients with both cardiac and non-cardiac conditions, further research is required to determine whether the search for false-negative cases should be restricted to cases coded within the ICD rubrics for ischaemic heart disease or chest pain.

Finally, the International expert panel made no recommendations about the AHA categories of MI (Definite, Probable, Possible) that should be included in epidemiological studies. Definite plus Probable MI in the AHA classification appears to be comparable to "Definite MI" used as the main non-fatal event in the MONICA Project [16] and appears to be similar to the definition of MI used in the Atherosclerosis Risk in Communities (ARIC) study [17]. Whether Possible MI (or any subset thereof) should also be included needs to be determined.

### What is already known on this subject

• Troponin tests increase counts of myocardial infarction (MI) in administrative data compared with counts based on traditional cardiac biomarkers.

• Long-term trends in MI based on traditional biomarkers are declining, whereas those based on troponin tests are increasing.

• Increased counts of MI associated with troponin tests using revised criteria for MI are only partly reflected in administrative data, possibly because physicians are reluctant to diagnose MI on the basis of relatively small increases in troponin levels.

### What this study adds

• This is the first population-wide study to explore the practical issues of implementing the revised criteria for myocardial infarction (MI) for use in epidemiological studies published by the American Heart Association (AHA) International panel in 2003. It shows that for trend analysis at least, the AHA criteria are flawed because they do not recognise variation in troponin assay thresholds in different laboratories or changes in diagnostic thresholds over time. This problem will increase with the development of even more sensitive troponin assays.

• Studies of trends in MI (as exemplified by the WHO MONICA Project) have a basic requirement for unchanging objective criteria. Our study identifies further work that is required to standardise troponin testing for analysis of trends, particularly at the International level, if the AHA criteria are to have any utility.

• Future epidemiological studies using AHA criteria will need to recognise the large increase in false-negative cases of MI associated with the large general increase in the prevalence of troponin testing in cases with other cardiac conditions or chest pain.

• Since the introductions of troponins, routinely collected hospital statistics no longer reflect true underlying trends in MI.

## Abbreviations

AHA: American Heart Association; HMDC: Hospital Morbidity Data Collection; MI: myocardial infarction; MI-HMDC: MI coded in the HMDC; MI-AHA: MI classified by the AHA criteria (including troponin tests); MI-AHA_ck_: MI classified by the AHA criteria using traditional cardiac biomarkers only (excluding troponin tests); Positive MI: AHA Definite or Probable MI; Any MI: AHA Definite, Probable or Possible MI; UAP: unstable angina pectoris

## Competing interests

The authors declare that they have no competing interests.

## Authors' contributions

All authors read and approved the final manuscript. MH, MK, JF, JR, PS, JH are chief investigators for this study. They designed the study, provided epidemiological (MH, JF), clinical (JR, JH, PS) and statistical (MK) guidance, and assisted in preparation and review of the manuscript. MH was the lead author for sections on Background, Discussion and Conclusions. FS implemented the study, managed research staff and the collection of data, carried out the data mining and all of the statistical analyses, created the Endnote library of references, and prepared the manuscript. FS was the lead author for the Methods and Results sections of the manuscript, and reviewed the Background, Discussion and Conclusions. SR prepared the data files, merging and linking laboratory data with administrative data, assisted with identifying and sampling of the validation population and sample, and reviewed the manuscript. PB prepared the list of variables for data collection, trained the research staff in the data collection techniques from medical notes, and reviewed the manuscript.

## Pre-publication history

The pre-publication history for this paper can be accessed here:

http://www.biomedcentral.com/1471-2261/11/35/prepub

## Supplementary Material

Additional file 1 **Methods: Additional Detail**. Provides additional information on the selection of the validation sample, and the classification of biomarkers, ECGs and symptoms using AHA definitions. Includes two additional tables associated with methods and results sections of the main text, including Table 1 from the AHA 2003 classification [12] showing counts from our validation samples. Click here for file
