# Supplementary material for: Can we monitor heart attack in the troponin era: evidence from a population-based cohort study
Source: BMC Cardiovasc Disord. 2011 Jun 24;11:35. doi: 10.1186/1471-2261-11-35 (PMC3224105; doi:10.1186/1471-2261-11-35)
Supplement: Additional file 1 — Methods: Additional Detail. Provides additional information on the selection of the validation sample, and the classification of biomarkers, ECGs and symptoms using AHA definitions. Includes two additional tables associated with methods and results sections of the main text, including Table 1 from the AHA 2003 classification [12] showing counts from our validation samples. [file 1471-2261-11-35-S1.PDF]

Can We Monitor Heart Attack In The Troponin Era: Evidence From A Population-Based Cohort Study

METHODS: ADDITIONAL DETAIL

Selection of validation sample

For each episode of care, we determined whether each biomarker test result was positive, negative, equivocal or missing, based on the diagnostic thresholds of each hospital laboratory in each year. The thresholds were lower in 2003 than in 1998 and were different in each hospital, with some hospitals changing assays and thresholds during either year. A positive troponin result was one that was above its upper level of normal, whilst a positive CK or CK-MB was a result of >2 times its upper level of normal. If different biomarker tests were performed we gave preference to troponin then CK-MB then CK when determining the overall biomarker result for that episode of care. Episodes of care were stratified into validation population sub-groups based on the hierarchical diagnosis, admission type (booked or emergency from the HMDC) and overall biomarker result, and a random sample selected from each stratum for validation (see Additional File 1, Table S1).

Classification of biomarkers, ECGs and symptoms using AHA definitions

Using AHA definitions [1], multiple biomarker results were classified overall as diagnostic, equivocal, normal or missing, with normal indicating that all biomarker results were negative. As the AHA definitions are not completely clear on how to handle various scenarios of biomarker tests, our classification algorithm was based on our own interpretation. For example, we recorded biomarkers as diagnostic instead of equivocal in cases where there was a single positive troponin test and an adequate set of CK tests that were also positive, even though troponin ranks higher than CK as an individual test.

ECGs were coded by a single trained coder using the Minnesota coding system [2], and classified as evolving diagnostic, positive, non-specific or normal/other ECG finding according to the AHA definitions for ECGs [1]. Symptoms were classified either as: (i) present if the medical notes recorded typical cardiac ischaemic pain (chest, arm, jaw), or congestive heart failure or cardiogenic shock as acute complications of the cardiac event, or (ii) absent if only atypical symptoms (nausea, pallor, sweating, collapse, breathlessness) or no symptoms were stated in the notes [1].

References

1. Luepker RV, Apple FS, Christenson RH, *et al.* Case definitions for acute coronary heart disease in epidemiology and clinical research studies: a statement from the AHA Council on Epidemiology and Prevention; AHA Statistics Committee; World Heart Federation Council on Epidemiology and Prevention; the European Society of Cardiology Working Group on Epidemiology and Prevention; Centers for Disease Control and Prevention; and the National Heart, Lung, and Blood Institute. *Circulation* 2003;**108**:2543-9.

2. Prineas RJ, Crow RS, Blackburn H. The Minnesota code manual of electrocardiographic findings: standards and procedures for measurement and classification. Boston, Massachusetts: Wright-PSG, 1982.

SUPPLEMENTARY TABLES

Table S1 Sampling scheme and sampling fractions for selection of validation sample of non-fatal cases in 1998 and 2003 based on type of admission, hierarchical cardiovascular discharge diagnosis and cardiac biomarker results

Validation sample groups (strata)	Sampling fraction (%)	Final count
**1998 (population 3522; sample 1456)**		
non-fatal MI (with or without biomarker) [a]	49.79	707
non-fatal UAP (no or -ve biomarker) [a]	32.77	602
non-fatal UAP (+ve biomarker) [a]	56.90	99
other angina (+ve biomarker) [a]	50.00	5
other cardiac diagnoses (+ve biomarker, emergency) [b]	53.09	43
**2003 (population 3297; sample 1108)**		
non-fatal MI (with or without biomarker) [a]	19.95	315
non-fatal UAP (no test, -ve or equivocal biomarker) [a]	23.82	289
non-fatal UAP (+ve biomarker) [a]	99.64	279
other cardiac diagnoses (+ve biomarker, emergency) [b]	97.60	225

-ve biomarker: negative (normal) biomarker test; +ve biomarker: positive biomarker; equivocal biomarker: result lies in the middle range of a 3-tier system (normal, equivocal, positive) that some hospitals used in 2003 to indicate minor myocardial damage; UAP: unstable angina pectoris.

[a] Includes booked and emergency admissions.

[b] Other cardiac diagnoses are those from other angina to chest pain (see Table 1 in main text).

Table S2 Distribution of AHA classification of myocardial infarction for 1998 and 2003 non-fatal population estimates based on symptoms, biomarkers and ECG changes

**1998**	**Cardiac symptoms or signs present**				**Cardiac symptoms or signs absent**				**Row Total**
	**Biomarker classification**				**Biomarker classification**				
**ECG classification**	Diagnostic	Equivocal	Missing	Normal	Diagnostic	Equivocal	Missing	Normal	
Evolving diagnostic	194	40	0	31	42	4	0	12	**323**
Positive	182	30	0	68	54	14	0	8	**356**
Non-specific	247	76	11	388	105 [a]	19	13	82	**941**
Normal/Other	317	136	37	962	118 [b]	59	76	197	**1902**
**Column total**	**940**	**282**	**48**	**1449**	**319**	**96**	**89**	**299**	**3522**

**2003**	**Cardiac symptoms or signs present**				**Cardiac symptoms or signs absent**				**Row Total**
	**Biomarker classification**				**Biomarker classification**				
**ECG classification**	Diagnostic	Equivocal	Missing	Normal	Diagnostic	Equivocal	Missing	Normal	
Evolving diagnostic	130	87	0	17	44	25	0	9	**312**
Positive	160	115	0	54	71	0	0	4	**404**
Non-specific	374	76	8	332	194 [c]	30	5	60	**1079**
Normal/Other	367	67	6	654	240 [d]	24	45	99	**1502**
**Column total**	**1031**	**345**	**14**	**1057**	**549**	**79**	**50**	**172**	**3297**

Definite MI   Probable MI   Possible MI   Not MI

AHA: American Heart Association; MI: myocardial infarction.
Cell counts represent the distribution of AHA classification of MI (Definite, Probable, Possible, Not MI) as shown in Table 1 of Luepker et al [1].
Number of cases downgraded to Possible MI due to absence of diagnostic troponin test: [a] 34 of 105 cases, [b] 32 of 118 cases, [c] 36 of 194 cases, and [d] 28 of 240 cases.
